# A Novel Multi-Preprocessing Integration Method for the Qualitative and Quantitative Assessment of Wild Medicinal Plants: *Gentiana rigescens* as an Example

**DOI:** 10.3389/fpls.2021.759248

**Published:** 2021-10-08

**Authors:** Zhimin Liu, Tao Shen, Ji Zhang, Zhimin Li, Yanli Zhao, Zhitian Zuo, Jinyu Zhang, Yuanzhong Wang

**Affiliations:** ^1^Medicinal Plants Research Institute, Yunnan Academy of Agricultural Sciences, Kunming, China; ^2^School of Agriculture, Yunnan University, Kunming, China; ^3^College of Chemistry, Biological and Environment, Yuxi Normal University, Yuxi, China

**Keywords:** wild *Gentiana rigescens*, multi-block data analysis, spectroscopy, pre-processing, sequential preprocessing through orthogonalization (SPORT)

## Abstract

Until now, the over-exploitation of wild resources has increased growing concern over the quality of wild medicinal plants. This led to the necessity of developing a rapid method for the evaluation of wild medicinal plants. In this study, the content of total secoiridoids (gentiopicroside, swertiamarin, and sweroside) of *Gentiana rigescens* from 37 different regions in southwest China were analyzed by high performance liquid chromatography (HPLC). Furthermore, Fourier transform infrared (FT-IR) was adopted to trace the geographical origin (331 individuals) and predict the content of total secoiridoids (273 individuals). In the traditional FT-IR analysis, only one scatter correction technique could be selected from a series of preprocessing candidates to decrease the impact of the light correcting effect. Nevertheless, different scatter correction techniques may carry complementary information so that using the single scatter correction technique is sub-optimal. Hence, the emerging ensemble approach to preprocessing fusion, sequential preprocessing through orthogonalization (SPORT), was carried out to fuse the complementary information linked to different preprocessing methods. The results suggested that, compared with the best results obtained on the scatter correction modeling, SPORT increased the accuracy of the test set by 12.8% in qualitative analysis and decreased the RMSEP by 66.7% in quantitative analysis.

## Introduction

The medicine plant, commonplace throughout human history, is an indispensable part of modern human medicine and traditional medicine (Balunas and Kinghorn, 2005; Ramawat et al., 2009; Bai et al., 2019). It has met the health care need of three-quarters of the population of the world, which is of great importance to save human life and promote economic development, especially for those who live in rural areas (He et al., 2018). As a matter of fact, concomitant with the rise of commercial demand and overharvesting, high-value medicinal plants will face an increased risk of complete extinction due of course to the fact that wild collection is the main source for the supplement of medicinal plants (Chi et al., 2017; Cunningham et al., 2018; Applequist et al., 2020; Kunwar et al., 2020). For example, *Gentiana rigescens* Franch has become one of the 10 most important endangered medicinal plants in Yunnan province since 2002. Furthermore, wild *G. rigescens* has also been listed as a class III protected species of wild medicinal herbs in the “List of Species of Wild Medicinal Herbs under State Key Protection.”

Wild *G. rigescens*, a representative medicine plant, is mainly grown in the Yunnan-Guizhou Plateau (southwest China) (Smith Olsen and Overgaard Larsen, 2003). The root tissue of wild *G. rigescens* is rich in flavonoids, alkaloids, triterpenoids, and iridoids (Pan et al., 2016). Note that the secoiridoids (loganin, swertiamarin, gentiopicroside, and sweroside) are the main bioactive ingredients, which are responsible for several pharmacological activities, such as hepatoprotective, cholagogue, anti-oxidant, and anti-cancer properties (Pan et al., 2016). Nevertheless, the particular mountain area and low latitude plateau in southwest China causes the difference in the content of secoiridoids, which in turn affects the multi-component coordination exerting the multi-channel and multi-target pharmacological action (Liu et al., 2020a). Furthermore, with the increasing demand for high-quality wild *G. rigescens*, the contemporary phenomenon of origin fraud and using low-quality *G. rigescens* as a substitute has been frequently observed, which greatly influences the supplement of world markets and the trust of consumers. Therefore, these factors have led to the necessity of applying a reliable approach for the qualitative and quantitative assessment of wild *G. rigescens*.

Fourier transform infrared (FT-IR) is regularly used for both qualitative and quantitative analysis since it has the advantages of being the simplest sampling method, is non-destructive, and has a low analysis cost (Liu et al., 2020b). New applications of FT-IR technique have been demonstrated and published daily in the fields of source and type authentication, fraud detection, and estimation of ingredient proportion, etc. (Bunaciu et al., 2011; Li et al., 2020; Liu et al., 2021a). FT-IR, as with other vibrational spectra technologies, deeply suffered from spurious sources of variability in the signal brought by additional unwanted interactions of light with the samples (Pei et al., 2019). Along with the evolution of computer science, scatter correction methods using mathematical techniques have developed significantly to overcome the light scatter effects encountered with FT-IR. Therefore, FT-IR spectroscopy, in combination with scatter correction techniques, has been widely applied, such as in reports involving wild *Boletus edulis* (Li et al., 2017; Li and Wang, 2018), *Radix Astragali* (Yang et al., 2021), *and Panax notoginseng* (Li et al., 2018). Nevertheless, the data modeling in all these studies can easily become sub-optimal since only one single scatter correction technique could be selected from a shortlist of potential candidates (Mishra et al., 2020a; Roger et al., 2020a).

Sequential preprocessing through orthogonalization (SPORT), a novel ensemble approach to the preprocessing fusion technique, takes inspiration from sequential and orthogonalized partial least-squares (SO-PLS). In fact, SPORT could lead to a boosting procedure by using SO-PLS to fuse several scatter correction techniques, since data processed with different scatter correction methods carry at least partially complementary information (Mishra et al., 2020a, 2021c). Recent momentum tends to indicate that the use of traditional single scatter correction methods has fallen out of favor in the emerging ensemble approaches to preprocessing fusion, such as reports involving wheat kernels (Mishra et al., 2021b), minced pork meat (Mishra et al., 2021d) fresh fruits (Mishra et al., 2020c), and fuel (Mishra et al., 2021a).

The goal of this study was to build a chemical method using multi-preprocessing integration for the qualitative and quantitative assessment of wild *Gentiana rigescens*. To this aim, the content of total secoiridoids of *Gentiana rigescens* (37 batches) was determined by high performance liquid chromatography (HPLC). Furthermore, SPORT was carried out to integrate the complementary information linked to FT-IR spectra processed by different preprocessing techniques for tracing the geographical origins (331 individuals) and predicting the content of secoiridoids (273 individuals). With respect to the quality assessment of wild medicinal plants, the approach of using multi-preprocessing integration in this paper not only provides an effective data fusion strategy without any extra instrument/sample information but also means that users do not need to select the best preprocessing methods.

## Materials and Methods

### Material and Reagents

**FIGURE 1:**
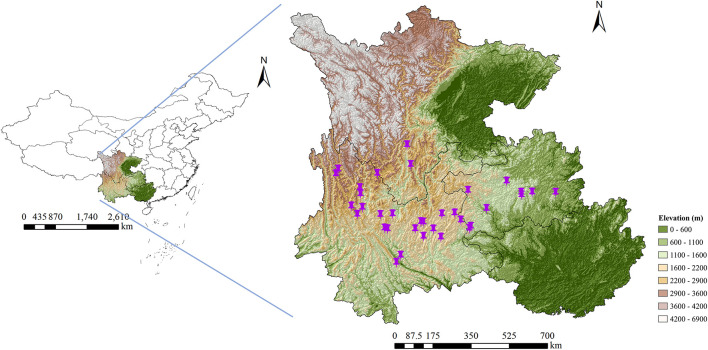
The geographical location of wild *G. rigescens* samples.

The wild *G. rigescens* samples in the blooming period were collected from Yunnan, Guizhou, and Sichuan provinces (southwest China). Based on the commitment to ensure sustainable utilization, 331 individuals of 35 batch samples were utilized for the qualitative analysis (the discrimination of geographical origin). Among them, 273 individuals were utilized for the qualitative analysis (the determining of total secoiridoids content). Note that the sampling locations covered the main distribution areas of *G. rigescens* (Figure 1). All samples used in this study were authentic and were gained directly by wild foraging. Detailed information on this is described in Table 1. All fresh root tissues of *G. rigescens* were washed and dried at 50? to a constant weight in the oven. Samples were then ground to powder with 60 mesh and reserved in a PE zip-lock bag at room temperature for further analysis.

**TABLE 1:** The detail information of *G. rigescens*.

No	Location	Latitude	Longitude	Elevation (m)	Number
DZ	Tuanjie Xishanxian Kunming Yunnan	N25°3′22″	E102°32′41″	2114	10
	Yiliang Kunming Yunnan	N25°02′57″	E103°17′39″	1817	10
	Shilin Kunming Yunnan	N24°42′47″	E103°36′19″	2144	10
	Baiyi Guandu Kunming Yunnan	N25°19′62″	E102°52′38″	2292	8
	Aziying Guandu Kunming Yunnan	N25°20′53″	E102°47′12″	2180	10
	Xinping Yuxi Yunnan	N25°20′53″	E102°47′12″	2180	10
	Chengjia Yuxi Yunnan	N23°58′01″	E101°56′57″	2016	9
	Yuanjiang Yuxi Yunnan	N24°44′53″	E102°53′22″	2643	10
	Luoping Qujing Yunnan	N23°40′00″	E101°46′13″	2150	10
	Zhanyi Qujing Yunnan	N25°39′57″	E103°38′49″	2044	9
	Fucun Fuyuan Qujing Yunnan	N25°25′04″	E104°25′43″	1954	9
	Zhongan Fuyuan Qujing Yunnan	N25°42′22″	E104°10′27″	2072	9
	Dayao Chuxiong Yunnan	N25°40′06″	E101°36′44″	1804	7
	Nanhua Chuxiong Yunnan	N25°05′05″	E101°16′51″	1969	10
	Yaoan Chuxiong Yunnan	N25°38′32″	E101°06′21″	2082	10
	Zixishan Chuxiong Yunnan	N25°03′32″	E101°24′03″	2105	10
	Fengjingqu Zixishan Chuxiong Yunnan	N25°23′02″	E101°22′05″	2100	10
	Lufeng Chuxiong Yunnan	N25°08′12″	E102°03′21″	1636	10
	Nanhua Chuxiong Yunnan	N25°05′34″	E101°16′51″	1946	10
DX	Yibin Dali Yunnan	N25°56′39″	E100°22′15″	2460	10
	Cangshan Dali Yunnan	N25°39′03″	E100°09′48″	2225	10
	Erhai Dali Yunnan	N25°59′44″	E99°54′46″	2350	10
	Heqing Dali Yunnan	N26°29′49″	E100°17′00″	2505	10
DXB	Yulong Lijiang Yunnan	N26°45′21″	E100°16′28″	2256	10
	Nilang Lijiang Yunnan	N27°20′25″	E100°59′01″	3219	10
	Pantiange Weixi Diqing Yunnan	N27°19′45″	E99°16′41″	2893	10
	Tacheng Weixi Diqing Yunnan	N27°31′05″	E99°22′10″	2520	10
GZ	Qishe Xingyi Guizhou	N25°09′41″	E104°50′24″	2158	9
	Baitaowan Xingyi Guizhou	N25°04′36″	E104°46′22″	2028	10
	Guanling Anshun Guizhou	N25°53′33″	E105°28′56″	1562	10
	Huangsi Fuquan Guizhou	N26°34′10″	E107°21′53″	1550	8
	Shuangliu Kaiyang Guiyang Guizhou	N27°01′35″	E106°19′09″	1600	7
	Zhanjie Qingzhen Guizhou	N26°38′22″	E104°43′26″	1590	9
	Jichang Longli Duyun Guizhou	N26°33′33″	E106°54′59″	1393	10
	Longjiangshan Longli Duyun Guizhou	N26°27′38″	E106°55′40″	1300	10
	Taigong Taijiang Guizhou	N26°33′36″	E108°19′45″	1260	9
SC	Dajing Xichang Sichuan	N27°42′36″	E102°21′30″	2258	10
	Chengxiang Guanning Sichuan	N28°31′27″	E102°12′19″	1921	8

Acetonitrile and formic acid of HPLC were purchased from DikmaPure (Beijing, China). Water was purchased from Wahaha (Hangzhou, China). Reference standards (gentiopicroside, swertiamarin, and sweroside) were provided by the Control of Pharmaceutical and Biological Products (Beijing, China). The purity of the reference compound analyzed by HPLC-MS was more than 98%, and the structure is described in Supplementary Figure 1.

### Chromatographic Determination

In this experiment, the quantitative analysis was performed by an Agilent 1260 Infinity HPLC system equipped with a G13311C diode array detector (GL Sciences Company, Japan). In an attempt to acquire the best chromatographic conditions, the detection wavelength, mobile phases, column temperature, and the type of chromatographic column were studied. Finally, the column temperature was maintained at 35? and the chromatographic separation was performed on an Agilent Intersil-C18 column (150 mm × 4.6 mm, 5 um). The mobile phase consisted of 0.1% aqueous formic acid in water (A) and acetonitrile (B), and the following gradient was used: 5% B, for the first 5 min; 5–10% A, for 5–10 min; 10–26% B, for 10–26 min; 26–30% B, for 26–30 min. Column temperature was maintained at 30°C and the detective wavelength was set at 246 nm. The flow rate was kept at 0.3 ml/min and the injection volume was 10 ul. The detection wavelength was set as 241 nm.

### Mid-Infrared Spectra Acquisition

In this experiment, FT-IR spectra were obtained by a FT-IR Spectrometer (PerkinElmer, United States) equipped with a deuterate triglycine sulfate detector. For each sample, 1.5 ± 0.2 mg of powder was mixed with spectrometry grade KBr (100 mg) in the agate mortar. The parameters of spectra acquirement were 32 co-added scans, scanning range (4,000–4,00 cm^–1^), and resolution (4 cm^–1^). Each spectrum was scanned in three times, and the average spectra were calculated and used as the final result. Note that background interferences caused by H_2_O and CO_2_ should be eliminated before the scanning of the blank KBr.

### The Images Acquisition of Synchronous Two-Dimensional Correlation Spectra

In order to preliminarily validate whether the different processed MIR data contained complementary information, the generalized two-dimensional correlation spectra (2DCOS) algorithm was carried out to generate synchronous 2DCOS images for identifying the overlapping peaks more effectively by imposing external disturbance on the samples. The flowchart of the whole conversion process is described in Supplementary Figure 2. The dynamic spectral intensities are expressed as a column vector S at variable v when the spectra with an equal interval of perturbation t are measured at m steps.


(1) $$\text{s}\,\left(v\right)=\left[\begin{array}{c} s\,\left(v,t_{1}\right)\\ s\,\left(v,t_{2}\right)\\ .\\ .\\ .\\ s\,\left(v,t_{m}\right) \end{array}\right]$$

Hence, the synchronous two-dimensional correlation intensity Φ (***V****_1_*, ***V****_2_*) between ***V*_1_** and ***V*_2_** are expressed as:


(2) $$\Phi \,\left(v_{1},v_{2}\right)=\frac{1}{m-1}\,\text{S}\,{\left({\text{v}}_{1}\right)}^{T}\times \text{S}\,\left({\text{v}}_{2}\right)$$

### Data Analysis

#### Scatter Correction Methods

The collected FT-IR initial spectra data were influenced by many unwanted artifacts that made the data unsuitable for direct analysis. Of particular concern were the light scattering effects. These factors led to the necessity for applying the scatter correction approaches. In the present study, four scatter correction approaches, including multiplicative scatter correction (MSC) (Isaksson and Næs, 1988), standard normal variate (SNV) (Barnes et al., 1989), variables sorting for normalization (VSN) (Rabatel et al., 2020), and second derivative (SD) (Roger et al., 2020b), were carried out. All analyses were carried out in MATLAB 2017b (The MathWorks, Natick, United States).

#### Multivariate Statistical Analysis

In the present study, two multivariate analysis methods, including PLS-DA and PLSR, were carried out. PLS-DA, a widely applied discrimination algorithm, divided the multi-dimensional space into class-regions, hence, the under tested samples were assigned to one specific category. More detailed information can be found in Ruiz-Perez et al. (2020). Three parameters, including the specificity, sensitivity, and accuracy, were calculated in an attempt to estimate the PLS-DA model’s performance. PLSR, a typical regression approach, is commonly utilized for MIR data modeling (Wold et al., 2001). It can be carried out to transform the high-dimensional data into the subspace of latent variables (LVs) through maximizing the covariance of the MIR data with the predicting response variables (Kestens et al., 2008). In the present study, two parameters, including the correlation coefficient of validation (R^2^p) and root mean square error of validation (RMSEP), were calculated to estimate the PLSR model’s performance. The PLS-DA and PLSR were performed by SIMCA-P+ (Version 13.0, MKS Umetrics) software.

#### Sequential Preprocessing Through Orthogonalization

Sequential preprocessing through orthogonalization, an emerging ensemble approach to preprocessing fusion, was developed from sequential and orthogonalized partial least-squares (SO-PLS) modeling (Roger et al., 2020a). It takes a multi-block dataset as the input data to establish the model for discriminating the geographical origins and predicting the response variables by means of SO-PLS. Note that the multi-block dataset which consisted of the FT-IR data was processed by different preprocessing approaches. A schematic illustration of the SPORT method is described in Supplementary Figure 3. As can be seen, PLS was utilized to fit the actual response and the first block, meanwhile, the score of the first block (T1) was thus calculated. The second block was orthogonalized with T1, and the orthogonalized second block was then used to fit the residuals of response by PLS. Note that the number of produce iterations was identical to the number of the inputted blocks in the SPORT model. Finally, all possible combinations of LVs extracted from different blocks were tested to select the optimal combination of LVs which had the lowest root mean square error of cross-validation (RMSECV) for the further qualitative and quantitative analysis. SPORT was carried in MBA-GUI, freely available in Mishra et al. (2020b).

## Results and Discussion

### Quantitative Analysis of the Total Secoiridoids

In this experiment, the external standard approach was performed to quantify the content of total secoiridoids (gentiopicroside, sweroside, and swertiamarin). All standard solutions were diluted into different concentrations using methanol in an attempt to establish the regression equation. Finally, a good linear relationship was achieved (*r*^2^ > 0.9991) for three standard solutions (gentiopicroside, sweroside, and swertiamarin). Note that before sample determination, the reasonability of the quantitative analysis approach was tested by calculating the revealed standard deviations (RSDs) of precision, repeatability, stability, and recovery. Information detailing this is shown in Supplementary Tables 1, 2. The content of the total secoiridoids is shown in Supplementary Table 3. Furthermore, in an attempt to preliminarily analyze the quality difference of *G. rigescens* collected from different regions, the coefficient of variation was calculated. The coefficient of variation was identical to 25.2%, which indicated that the quality of *G. rigescens* was greatly varied. Hence, the further qualitative and quantitative assessment of the wild *G. rigescens* is of great significance for practical application in routine life.

### Raw Fourier Transform Infrared Spectra Analysis

**FIGURE 2:**
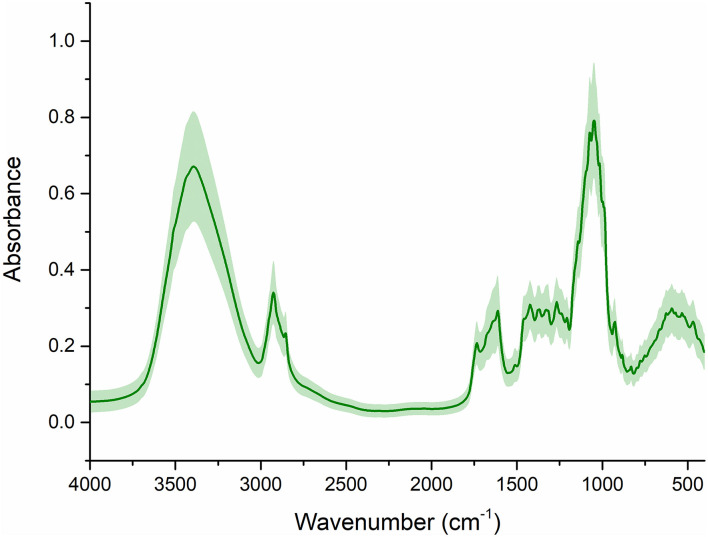
The raw FT-IR spectra. The line represents the average spectra of wild *G. rigescens*, and the shade around the average spectra represents the standard deviation.

The spectra obtained with FT-IR (Figure 2) exhibited eight main absorption bands (approximately 3400, 2928, 2857, 1615, 1427, 1375, 1260, and 1057 cm^–1^), which represented the different vibrational models of functional groups. In an attempt to gain insight into the spectral characteristics of *G. rigescens*, they were interpreted as follows: (i) the first overtone O-H stretching resulted in a peak at 3,400 cm^–1^, (ii) methylene asymmetry caused by esters can be seen at around 2,928 and 2,857 cm^–1^; (iii) a band at 1,615 cm^–1^ resulted in the asymmetric stretch of the C-C bond; (iv) a peak at 1,427 cm^–1^ was assigned to the asymmetric bending vibration of -CH_3_; (v) a peak at 1,375 cm^–1^ represented the bending vibration of -CH_3_ caused by esters, and (vi) peaks at 1,075 cm^–1^ were attributed to C-OH or C-O stretching (Zhao et al., 2019; Wang et al., 2020).

Nevertheless, it was apparent that the IR signals exhibited a similar trend, in particular in terms of the eight main absorption bands. Note that model performance, to the best of our knowledge, is affected by the unwanted artifacts in traditional modeling, in which light scattering effects are of particular concern. Therefore, it was of great importance to use scatter correction techniques for further analysis.

### The Processed Fourier Transform Infrared Spectra Analysis

**FIGURE 3:**
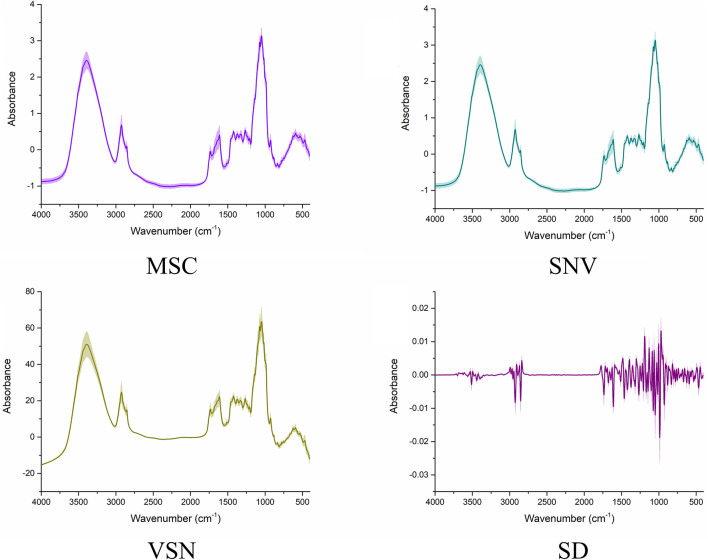
The FT-IR spectra processed by the four scatter correction techniques. The line represents the average spectra of wild *G. rigescens*, and the shade around the average spectra represents the standard deviation.

**FIGURE 4:**
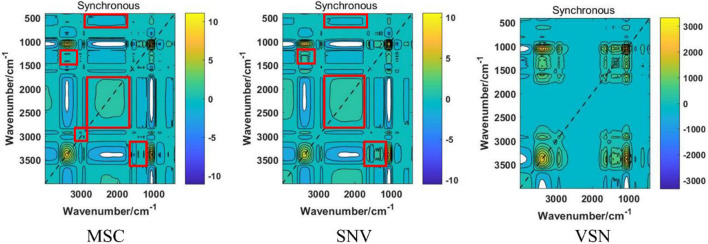
The reprehensive synchronous 2DCOS images of three preprocessing methods, including MSC, SNV, and VSN. The regions in the red boxes reflect the significant differences between MSC and SNV.

In the present study, four scatter correcting techniques, including MSC, SNV, VSN, and SD, were utilized to process the FT-IR spectra (Figure 3). From the intuitive identification approach view, standard deviation of the processed spectra was significantly decreased, especially if compared with the raw spectra. Hence, the scattering effects were reduced to different degrees in the four processed spectra. Note that SD significantly changed the structure of the data matrixes, which indicated there might be complementary information among SD and the other preprocessing methods. Nevertheless, there was no difference among MSC, SNV, and VSN according to one-dimensional (1D) linear spectra. Hence, 2DCOS was carried out to convert the 1D linear spectra into 2DCOS images to verify the necessity of using MSC, SNV, and VSN in SPORT. As can be seen from the 2DCOS images (Figure 4), there were differences in the synchronous 2DCOS images among MSC, SNV, and VSN, especially in terms of the intensity of the auto-peaks and cross-peaks. Hence, there might be complementary information among MSC, SNV, and VSN. Taking together, the data processed by the four scatter correction techniques might contain complementary information. Hence, the fusion of the above scatter correction techniques for the further qualitative and quantitative analysis of wild *G. rigescens* was significant.

### The Qualitative Assessment of Wild *G. rigescens*

In an attempt to establish a reliable discriminant model, 331 samples were divided into 182 samples (training set) and 91 samples (test set) by the Kennard-Stone (KS) algorithm. After that, five PLS-DA models were established based on the raw FT-IR spectra and four processed FT-IR spectra, and 7-fold cross-validation was carried out to determine the optimal number of LVs. Subsequently, SPORT was carried out to integrate the complement information linked to different preprocessing techniques for the qualitative assessment of wild *G. rigescens*. Note that although the input order could influence how the model is explained, it could not obviously affect the classification result of the model (Biancolillo et al., 2021). Hence, the order of the preprocessing methods, in the present study, was MSC, SNV, VSN, and SD. The selected result is shown in Supplementary Figure 4A. As can be seen, 9 LVs, 10 LVs, and 10 LVs were extracted in data processed by MSC, SNV, and SD, respectively. Note that the VSN block did not provide any complementary information, which indicated that all relevant information might already be modeled by earlier blocks. This is a very interesting observation. Finally, the PLS-DA model was built using the fused data matrix (361 samples × 29 LVs), and further applied to the test set.

The parameters, classification results, and confusion matrixes of the PLS-DA models are depicted in Supplementary Tables 4, 5 and Table 2. Furthermore, a 200-iteration permutation test was carried out to validate the fitting degree of the PLS-DA models, and the result showed that there was no overfitting in all the PLS-DA models (Supplementary Figure 5). In an attempt to better compare the classification performance of the PLS-DA models, the accuracy of test set is depicted in Supplementary Figure 6. As can be seen, scatter correction methods improved the classification performance to some extent, especially when compared with the raw spectral model. SD, the optimal scatter correction method in this study, increased the accuracy of the test set by 31.6%. It might be worth noting that the PLS-DA model based on SPORT, compared with the raw spectral model, increased the accuracy of the test set by 48.5%. Hence, SPORT could effectively fuse the complement information of different scatter correction techniques, which is of great importance for the qualitative analysis in other medicinal plants.

**TABLE 2:** The classification parameters of PLS-DA models.

	Training set				Test set		
		
	Classes	SEN	SPE	ACC	SEN	SPE	ACC
Raw	O1	0.92	0.64	0.78	0.90	0.58	0.74
	O2	0.74	0.79	0.78	0.85	0.73	0.74
	O3	0.58	0.81	0.78	0.64	0.75	0.74
	O4	0.61	0.83	0.78	0.39	0.84	0.74
	O5	0.73	0.79	0.78	0.71	0.75	0.74
	O1	0.88	0.51	0.69	0.85	0.45	0.65
MSC	O2	0.33	0.74	0.69	0.23	0.70	0.65
	O3	0.54	0.71	0.69	0.64	0.65	0.65
	O4	0.67	0.70	0.69	0.57	0.67	0.65
	O5	0.09	0.74	0.69	0.00	0.70	0.65
SNV	O1	0.88	0.57	0.72	0.89	0.48	0.68
	O2	0.41	0.76	0.72	0.31	0.73	0.68
	O3	0.62	0.74	0.72	0.64	0.69	0.68
	O4	0.67	0.74	0.72	0.50	0.74	0.68
	O5	0.36	0.75	0.72	0.43	0.72	0.68
VSN	O1	0.94	0.66	0.8	0.97	0.48	0.72
	O2	0.81	0.80	0.8	0.85	0.71	0.72
	O3	0.46	0.84	0.8	0.36	0.77	0.72
	O4	0.72	0.83	0.8	0.46	0.80	0.72
	O5	0.45	0.83	0.8	0.14	0.77	0.72
SD	O1	0.89	0.75	0.82	0.93	0.76	0.85
	O2	0.89	0.81	0.82	0.85	0.85	0.85
	O3	0.54	0.85	0.82	0.79	0.85	0.85
	O4	0.83	0.82	0.82	0.75	0.87	0.85
	O5	0.45	0.86	0.82	0.57	0.87	0.85
SPORT	O1	0.99	0.99	0.99	0.98	0.94	0.96
	O2	1.00	0.99	0.99	1.00	0.95	0.96
	O3	1.00	0.99	0.99	0.86	0.97	0.96
	O4	0.98	0.99	0.99	0.96	0.96	0.96
	O5	1.00	0.99	0.99	0.86	0.97	0.96

*SEN, sensitivity; SPE, specificity; ACC, accuracy.*

### The Quantitative Assessment of Wild *G. rigescens*

In an attempt to develop a reliable calibration model, the 273 samples were divided into 182 (calibration set) samples and 91 (test set) samples using the KS algorithm. The FT-IR spectra distribution of the calibration set and test set is displayed in Supplementary Figure 7. After that, the raw data and the four processed data were utilized to establish PLSR models, and the LVs selection method was consistent with PLS-DA. Subsequently, SPORT was performed to integrate the complement information linked to different preprocessing techniques for the quantitative assessment of wild *G. rigescens*. The fusion order of the scatter correction methods and the selection of an optical number of LVs were consistent with the qualitative analysis SPORT model, and the selected result is shown in Supplementary Figure 4B. Note that 10 LVs and 10 LVs were, respectively, extracted from data processed by MSC and SD. Thus, a total of 20 LVs were used as the input data to establish the SPORT model for further quantitative analysis.

**FIGURE 5:**
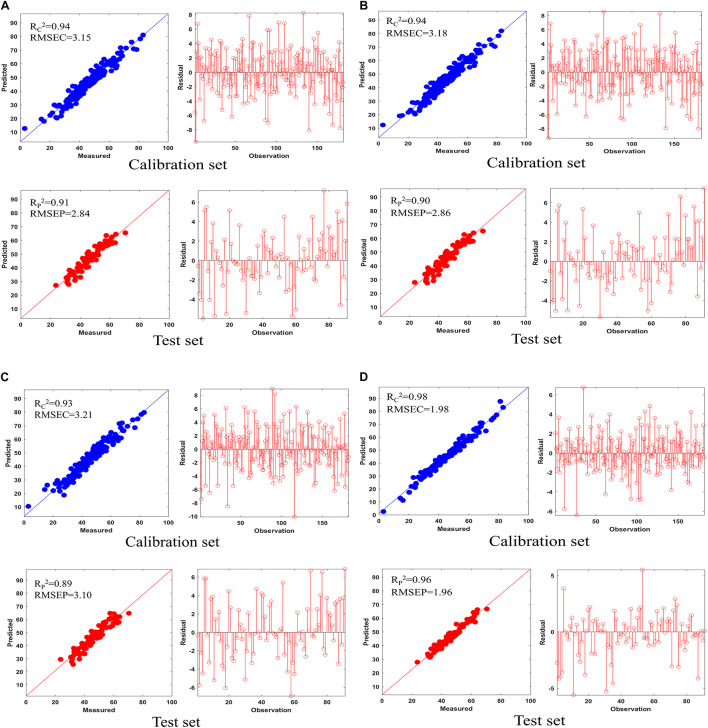
The results of five PLSR models on the calibration set and test set and their residual plot. **(A)** MSC; **(B)** SNV; **(C)** VSN; **(D)** SD.

**FIGURE 6:**
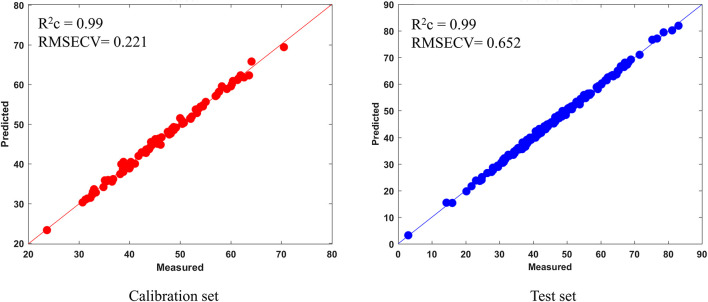
The results of the SPORT model on the calibration set and test set.

The prediction result and the residual plot of the PLSR models are described in Figure 5. As can be seen, the scatter correction methods improved the predictive performance, such as fit degree and residuals. After inspection of the parameters of the PLSR models (Table 3), the optimal model was obtained from the data processed by SD, especially when compared to the PLSR model established on raw data. Specifically, the SD approach increased R^2^p by 7.9% and decreased the RMSEP by 44.8%. On this basis, the results also indicated that SPORT, compared with the SD approach, significantly improved the predictive performance, especially in terms of the residual error (Figure 6). Specifically, the SPORT method increased R^2^p by 11.8% and decreased the RMSEP by 81.6%. In conclusion, when the qualitative analysis model was established based on a single scatter correction technique, the predictive performance was good, but less superior than that of the SPORT method.

**TABLE 3:** The parameters of the quantitative analysis model.

Methods	LVs	R_c_^2^	RMSECV	R_p_^2^	RMSEP
Raw	13	0.93	3.422	0.89	3.551
MSC	13	0.94	0.310	0.91	2.837
SNV	3	0.94	0.309	0.90	2.860
VSN	13	0.93	0.304	0.89	3.103
SD	8	0.98	0.292	0.96	1.959
SPORT	MSC (10) SD (10)	0.99	0.221	0.99	0.652

## Discussion

Generally, the scatter correction technique is required to remove light scattering effects so that the “true” chemically related spectra data can be underlined to obtain a high-quality model. With respect to the overall assessment of *Gentiana rigescens*, the different scatter correct techniques increased different extents of the model performance in the qualitative and quantitative analysis. Hence, the scatter correction technique might remove specific scattering effects while still leaving a part of different scattering effects. After the inspection of Tables 2, 3, the best result was provided by the ensemble approach to preprocessing fusion (SPORT), which indicated that SPORT could remove the scattering effect that would be left behind by using only one technique. Furthermore, in current industrial trends, the SPORT method, compared with the previous studies, also presented excellent advantages, in terms of the selection of an optimal preprocessing method and the fusion of complementary information.

The exploration of the optimal preprocessing method or their combination has attracted most attention in qualitative and quantitative analysis of food, and several methods have emerged one after another, such as the full factorial Design of Experiments (DoE)-based ensemble, in which all the possible combinations of preprocessing are utilized and the equality PLS models are calculated to select the optimal preprocessing combination, as in a report involving corn (Bian et al., 2020). Nevertheless, the above DoE-based selection method has two major limitations in its routine application: the first one is that it does not provide insight into what new information of each preprocessing method carried, and the second one is that exploring all the possible combinations of preprocessing is significantly time-consuming, which is not consistent with the rule of routine application. The emerging preprocessing fusion approach (SPORT) used in the present study could contribute to bringing in a new way of exploring the complementary information of different preprocessing methods. As a matter of fact, 9 LVs, 10 LVs, and 10 LVs were extracted in data processed by MSC, SNV, and SD, respectively, in terms of qualitative analysis, and 10 LVs and 10 LVs were, respectively, extracted from data processed by MSC and SD in terms of quantitative analysis. However, the number of LVs extracted in the qualitative and quantitative analysis was a bit on the high side. As the spectral datasets with a high number of spectral bands are complex, the upper number of LVs extracted in each block was limited in SPORT. Hence, in order to overcome this drawback, the number of LVs is one of the topics for future research in SPORT. Furthermore, the emerging preprocessing fusion approach also negates the need to select the best preprocessing methods and their combination on the part of the user, which is of great importance to routine application.

Data fusion, including low-level data fusion, mid-level data fusion, and high-level data fusion, was presented as a promising strategy for the quality assessment of medicinal plants, such as in reports involving *Polygonatum kingianum* (Zhang et al., 2021), *Amomum tsao-ko* (Liu et al., 2021b), and *Eucommia ulmoides* (Wang et al., 2020). Indeed, two very recent studies used data fusion in an attempt to integrate instrument/sample information for the rapid quality assessment of wild *G. rigescens*. The first one by Shen et al. (2020) used low- and mid-level data fusion strategies to integrate the different instrument information of wild *G. rigescens*, including near-infrared (NIR) and mid-infrared (MIR) spectroscopies, for the discrimination of the wild *G. rigescens* and its related species. The second one by Liu et al. (2020a) utilized a data fusion strategy to integrate homologous information of multi-part (root, stem, and leaf) samples for tracing the geographical origins of wild *Gentiana rigescens*. Although higher precision results were obtained in the above analysis, the increase in the amount of the instrument increases, and the cost will inevitably increase, resulting in an unsuitable application in routine life. In the present study, SPORT was carried out to significantly improve the performance of both qualitative and quantitative analysis models through integrating the complementary information linked to different preprocessing techniques. The results of this study indicated that SPORT could be defined as a possible alternative solution to a multi-platform integration strategy without any extra instrument/sample information. It is our expectation that SPORT can provide a low-cost strategy for the quality assessment of wild *G. rigescens*, and provide a certain reference value for further protection, development, and utilization of wild medicinal plants.

## Conclusion

In this study, the feasibility of qualitative and quantitative assessment of wild *G. rigescens* using multi-preprocessing integration was demonstrated by the standard PLS technique and SPORT technique. What we used is a reliable method with wide applicability, which can’t only be used as a possible solution to effectively fuse data without any extra instrument/sample information but also allows the selection of the best preprocessing method without user input. Hence, with respect to the qualitative and quantitative analysis of medicinal plants, the application of SPORT in FT-IR modeling is recommended, furthermore, the SPORT approach was not limited to FT-IR data, but it could be utilized to integrate multiple preprocessing techniques with any spectroscopic data. We expect that SPORT can provide a low-cost strategy for the quality assessment of wild *G. rigescens*, and provide a certain reference value for the further protection, development, and utilization of wild medicinal plants.

## Data Availability Statement

The original contributions presented in the study are included in the article/Supplementary Material, further inquiries can be directed to the corresponding author/s.

## Author Contributions

ZLiu contributed to conceptualization, writing – original draft, and data curation. TS contributed to investigation, methodology, resources, and software. JiZ contributed to investigation, resources, and formal analysis. ZLi contributed to investigation and resources. YZ contributed to resources and supervision. ZZ contributed to validation and visualization. JinZ and YW contributed to funding acquisition and project administration. All authors have read and approved the final manuscript.

## Conflict of Interest

The authors declare that the research was conducted in the absence of any commercial or financial relationships that could be construed as a potential conflict of interest.

## Publisher’s Note

All claims expressed in this article are solely those of the authors and do not necessarily represent those of their affiliated organizations, or those of the publisher, the editors and the reviewers. Any product that may be evaluated in this article, or claim that may be made by its manufacturer, is not guaranteed or endorsed by the publisher.

## References

[B1] Applequist W. L. Brinckmann J. A. Cunningham A. B. Hart R. E. Heinrich M. Katerere D. R. (2020). Scientists’ warning on climate change and medicinal plants. *Planta Med.* 86 10–18. 10.1055/a-1041-3406 31731314

[B2] Bai Y. Ochuodho T. O. Yang J. (2019). Impact of land use and climate change on water-related ecosystem services in Kentucky, USA. *Ecol. Indic.* 102 51–64. 10.1016/j.ecolind.2019.01.079

[B3] Balunas M. J. Kinghorn A. D. (2005). Drug discovery from medicinal plants. *Life Sci.* 78 431–441. 10.1016/j.lfs.2005.09.012 16198377

[B4] Barnes R. J. Dhanoa M. S. Lister S. J. (1989). Standard normal variate transformation and de-trending of near-infrared diffuse reflectance spectra. *Appl. Spectrosc.* 43 772–777. 10.1366/0003702894202201

[B5] Bian X. Wang K. Tan E. Diwu P. Zhang F. Guo Y. (2020). A selective ensemble preprocessing strategy for near-infrared spectral quantitative analysis of complex samples. *Chemometr. Intell. Lab. Syst.* 197:103916. 10.1016/j.chemolab.2019.103916

[B6] Biancolillo A. Preys S. Gaci B. Le-Quere J. Laboure H. Deuscher C. (2021). Multi-block classification of chocolate and cocoa samples into sensory poles. *Food Chem.* 340:127904. 10.1016/j.foodchem.2020.127904 32890856

[B7] Bunaciu A. A. Aboul-Enein H. Y. Fleschin S. (2011). Recent applications of Fourier transform infrared spectrophotometry in herbal medicine analysis. *Appl. Spectrosc. Rev.* 46 251–260. 10.1080/05704928.2011.565532

[B8] Chi X. Zhang Z. Xu X. Zhang X. Zhao Z. Liu Y. (2017). Threatened medicinal plants in China: distributions and conservation priorities. *Biol. Conserv.* 210 89–95. 10.1016/j.biocon.2017.04.015

[B9] Cunningham A. B. Brinckmann J. A. Bi Y. F. Pei S. J. Schippmann U. Luo P. (2018). Paris in the spring: a review of the trade, conservation and opportunities in the shift from wild harvest to cultivation of *Paris polyphylla* (Trilliaceae). *J. Ethnopharmacol.* 222 208–216. 10.1016/j.jep.2018.04.048 29727736

[B10] He J. Yang B. Dong M. Wang Y. (2018). Crossing the roof of the world: trade in medicinal plants from Nepal to China. *J. Ethnopharmacol.* 224 100–110. 10.1016/j.jep.2018.04.034 29705517

[B11] Isaksson T. Næs T. (1988). The effect of multiplicative scatter correction (msc) and linearity improvement in NIR spectroscopy. *Appl. Spectrosc.* 42 1273–1284.

[B12] Kestens V. Charoud-Got J. Bau A. Bernreuther A. Emteborg H. (2008). Online measurement of water content in candidate reference materials by acousto-optical tuneable filter near-infrared spectrometry (AOTF-NIR) using pork meat calibrants controlled by Karl Fischer titration. *Food Chem.* 106 1359–1365. 10.1016/j.foodchem.2007.01.081

[B13] Kunwar R. M. Adhikari Y. P. Sharma H. P. Rimal B. Devkota H. P. Charmakar S. (2020). Distribution, use, trade and conservation of *Paris polyphylla* Sm. in Nepal. *Glob. Ecol. Conserv.* 23:e1081. 10.1016/j.gecco.2020.e01081

[B14] Li Y. Shen Y. Yao C. Guo D. (2020). Quality assessment of herbal medicines based on chemical fingerprints combined with chemometrics approach: a review. *J. Pharm. Biomed. Anal.* 185:113215. 10.1016/j.jpba.2020.113215 32199327

[B15] Li Y. Wang Y. (2018). Synergistic strategy for the geographical traceability of wild *Boletus tomentipes* by means of data fusion analysis. *Microchem. J.* 140 38–46. 10.1016/j.microc.2018.04.001

[B16] Li Y. Zhang J. Li T. Liu H. Li J. Wang Y. (2017). Geographical traceability of wild boletus edulis based on data fusion of FT-MIR and ICP-AES coupled with data mining methods (SVN). *Spectrochim. Acta Part A* 177 20–27. 10.1016/j.saa.2017.01.029 28113137

[B17] Li Y. Zhang J. Wang Y. (2018). Ft-mir and nir spectral data fusion: a synergetic strategy for the geographical traceability of *Panax notoginseng*. *Anal. Bioanal. Chem.* 410 91–103. 10.1007/s00216-017-0692-0 29143877

[B18] Liu L. Zuo Z. Xu F. Wang Y. (2020b). Study on quality response to environmental factors and geographical traceability of wild *Gentiana rigescens* Franch. *Front. Plant Sci.* 11:1128. 10.3389/fpls.2020.01128 32793274 PMC7387691

[B19] Liu L. Zuo Z. Wang Y. Xu F. (2020a). A fast multi-source information fusion strategy based on ftir spectroscopy for geographical authentication of wild *Gentiana rigescens*. *Microchem. J.* 159:105360. 10.1016/j.microc.2020.105360

[B20] Liu Z. Yang M. Q. Zuo Y. Wang Y. Zhang J. (2021a). Fraud detection of herbal medicines based on modern analytical technologies combine with chemometrics approach: a review. *Crit. Rev. Anal. Chem.* 1–18. 10.1080/10408347.2021.1905503 33840329

[B21] Liu Z. Yang S. Wang Y. Zhang J. (2021b). Multi-platform integration based on nir and uv–vis spectroscopies for the geographical traceability of the fruits of *Amomum tsao-ko*. *Spectrochim. Acta Part A* 258:119872. 10.1016/j.saa.2021.119872 33957443

[B22] Mishra P. Biancolillo A. Roger J. M. Marini F. Rutledge D. N. (2020a). New data preprocessing trends based on ensemble of multiple preprocessing techniques. *Trends Anal. Chem.* 132:116045. 10.1016/j.trac.2020.116045

[B23] Mishra P. Marini F. Biancolillo A. Roger J. (2021a). Improved prediction of fuel properties with near-infrared spectroscopy using a complementary sequential fusion of scatter correction techniques. *Talanta* 223:121693. 10.1016/j.talanta.2020.121693 33303145

[B24] Mishra P. Nordon A. Roger J. (2021b). Improved prediction of tablet properties with near-infrared spectroscopy by a fusion of scatter correction techniques. *J. Pharm. Biomed. Anal.* 192:113684. 10.1016/j.jpba.2020.113684 33099114

[B25] Mishra P. Roger J. M. Rutledge D. N. Biancolillo A. Marini F. Nordon A. (2020b). MBA-GUI: a chemometric graphical user interface for multi-block data visualisation, regression, classification, variable selection and automated pre-processing. *Chemometr. Intell. Lab. Syst.* 205:104139. 10.1016/j.chemolab.2020.104139

[B26] Mishra P. Roger J. M. Rutledge D. N. Woltering E. (2020c). SPORT pre-processing can improve near-infrared quality prediction models for fresh fruits and agro-materials. *Postharvest Biol. Technol.* 168:111271. 10.1016/j.postharvbio.2020.111271

[B27] Mishra P. Rutledge D. N. Roger J. Wali K. Khan H. A. (2021c). Chemometric pre-processing can negatively affect the performance of near-infrared spectroscopy models for fruit quality prediction. *Talanta* 229:122303. 10.1016/j.talanta.2021.122303 33838766

[B28] Mishra P. Verkleij T. Klont R. (2021d). Improved prediction of minced pork meat chemical properties with near-infrared spectroscopy by a fusion of scatter-correction techniques. *Infrared Phys. Technol.* 113:103643. 10.1016/j.infrared.2021.103643

[B29] Pan Y. Zhang J. Shen T. Zhao Y. Zuo Z. Wang Y. Z. (2016). Investigation of chemical diversity in different parts and origins of ethnomedicine *Gentiana rigescens* Franch using targeted metabolite profiling and multivariate statistical analysis. *Biomed. Chromatogr.* 30 232–240. 10.1002/bmc.3540 26094855

[B30] Pei Y. Zuo Z. Zhang Q. Wang Y. (2019). Data fusion of fourier transform mid-infrared (MIR) and near-infrared (NIR) spectroscopies to identify geographical origin of wild *Paris polyphylla* var. Yunnanensis. *Molecules* 24:2559. 10.3390/molecules24142559 PMC6680555 31337084

[B31] Rabatel G. Marini F. Walczak B. Roger J. M. (2020). VSN: Variable sorting for normalization. *J. Chemometr.* 34:e3164. 10.1002/cem.3164

[B32] Ramawat K. G. Dass S. Mathur M. (2009). “The chemical diversity of bioactive molecules and therapeutic potential of medicinal plants,” in *Herbal Drugs: Ethnomedicine to Modern Medicine*, ed. Ramawat K. (Berlin: Springer), 7–32. 10.1007/978-3-540-79116-4_2

[B33] Roger J. M. Biancolillo A. Marini F. (2020a). Sequential preprocessing through orthogonalization (sport) and its application to near infrared spectroscopy. *Chemometr. Intell. Lab. Syst.* 199:103975. 10.1016/j.chemolab.2020.103975

[B34] Roger J. M. Boulet J. C. Zeaiter M. Rutledge D. N. (2020b). *Pre-Processing Methods, Reference Module in Chemistry, Molecular Sciences and Chemical Engineering.* Amsterdam: Elsevier.

[B35] Ruiz-Perez D. Guan H. Madhivanan P. Mathee K. Narasimhan G. (2020). So you think you can pls-da? *BMC Bioinformatics* 21:2. 10.1186/s12859-019-3310-7 33297937 PMC7724830

[B36] Shen T. Yu H. Wang Y. (2020). Discrimination of *Gentiana* and its related species using IR spectroscopy combined with feature selection and stacked generalization. *Molecules* 25:1442. 10.3390/molecules25061442 32210010 PMC7144467

[B37] Smith Olsen C. Overgaard Larsen H. (2003). Alpine medicinal plant trade and Himalayan Mountain livelihood strategies. *Geogr. J.* 169 243–254. 10.1111/1475-4959.00088

[B38] Wang C. Tang L. Li L. Zhou Q. Li Y. Li J. (2020). Geographic authentication of *Eucommia ulmoides* leaves using multivariate analysis and preliminary study on the compositional response to environment. *Front. Plant Sci.* 11:79. 10.3389/fpls.2020.00079 32140161 PMC7042207

[B39] Wold S. Sjöström M. Eriksson L. (2001). Pls-regression: a basic tool of chemometrics. *Chemometr. Intell. Lab. Syst.* 58 109–130. 10.1016/S0169-7439(01)00155-1

[B40] Yang J. Yin C. Miao X. Meng X. Liu Z. Hu L. (2021). Rapid discrimination of adulteration in Radix astragali combining diffuse reflectance mid-infrared fourier transform spectroscopy with chemometrics. *Spectrochim. Acta Part A* 248:119251. 10.1016/j.saa.2020.119251 33302218

[B41] Zhang J. Wang Y. Z. Yang M. Q. Yang W. Z. Yang S. B. Zhang J. Y. (2021). Identification and evaluation of *Polygonatum kingianum* with different growth ages based on data fusion strategy. *Microchem. J.* 160:105662. 10.1016/j.microc.2020.105662

[B42] Zhao Y. Yuan T. Zhang J. Wang Y. (2019). Geographic origin identification and rapid determination of four constituents of *Gentiana rigescens* by FTIR combined with chemometrics. *J. Chemometr.* 33:e3115. 10.1002/cem.3115

