# Supplementary material for: A Novel Multi-Preprocessing Integration Method for the Qualitative and Quantitative Assessment of Wild Medicinal Plants: Gentiana rigescens as an Example
Source: Front Plant Sci. 2021 Oct 8;12:759248. doi: 10.3389/fpls.2021.759248 (PMC8531481; doi:10.3389/fpls.2021.759248)
Supplement: Supplementary file 1 [file Data_Sheet_1.docx]

**A novel multi-preprocessing integration method for the qualitative and quantitative assessment of wild medicinal plants: *Gentiana rigescens* as an example**

Zhimin Liu ^a, b^, Tao Shen ^a^, JiZhang ^a^, Zhimin Li ^a^, Yanli Zhao ^a^, Zhitian Zuo ^a^, Jinyu Zhang ^a, b,^ *, Yuanzhong Wang^a,^ *

Affiliations:

^a^ Medicinal Plants Research Institute, Yunnan Academy of Agricultural Sciences, Kunming 650200, China

^b^ School of Agriculture, Yunnan University, Kunming 650500, China

***corresponding authors:**

Jinyu Zhang, Medicinal Plants Research Institute, Yunnan Academy of Agricultural Sciences, Kunming 650200, China. E-mail: jyzhang2008@126.com

Yuanzhong Wang, Medicinal Plants Research Institute, Yunnan Academy of Agricultural Sciences, Kunming 650200, China. E-mail: boletus@126.com

**Figure Captions:**

**Figure S1.** Chemical structures of the reference standards.

**Figure S2.** A flowchart of whole conversion processes of 2DOCS images through matlab2017b.

**Figure S3.** The schematic of SPORT for sequential fusion of pre-processing methods.

**Figure S4.** The number of selected LVs in SPORT model. (a) the qualitative analysis model; (b) the quantitative analysis model

**Figure S5.** Permutation test results of five PLS-DA models.
**Figure S6.** The accuracy of PLS-DA models.

**Figure S7.** The distribution curve of the calibration set and test set.


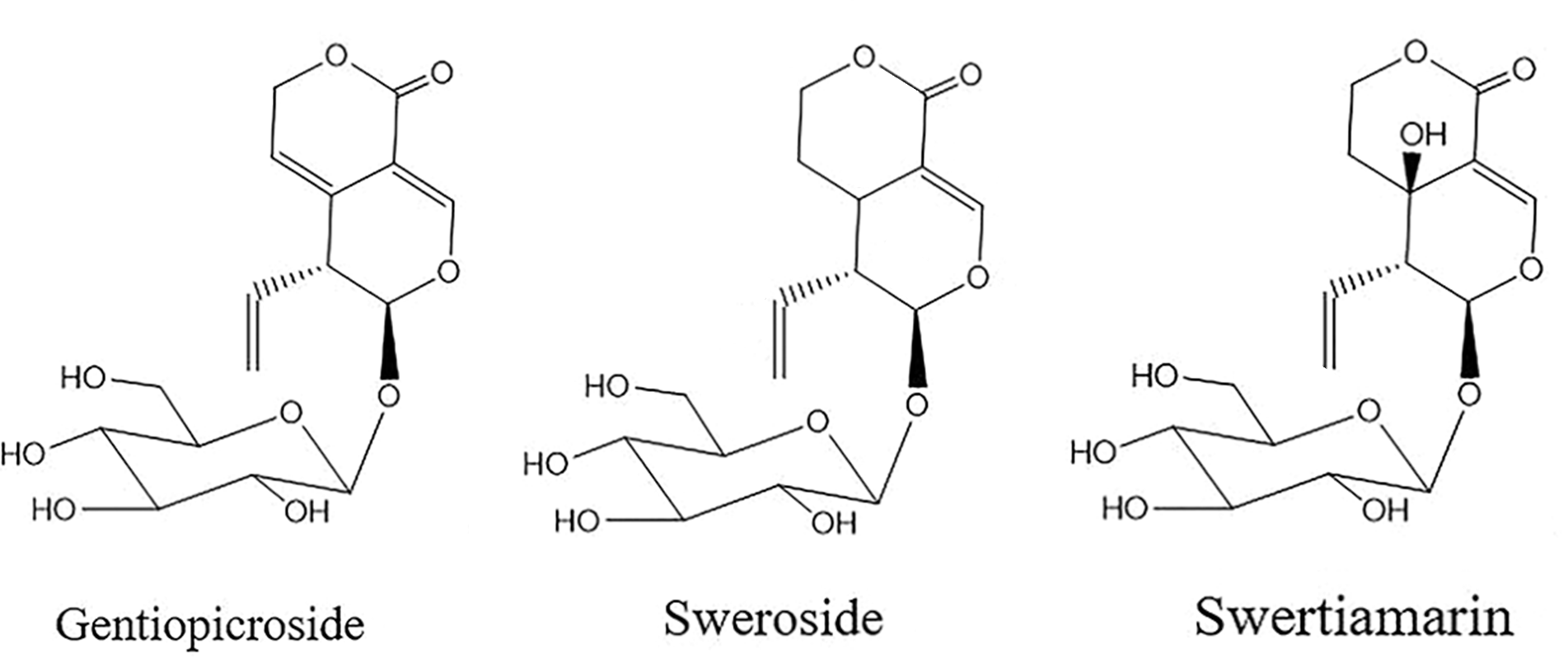


**Figure S1.** Chemical structures of the reference standards.


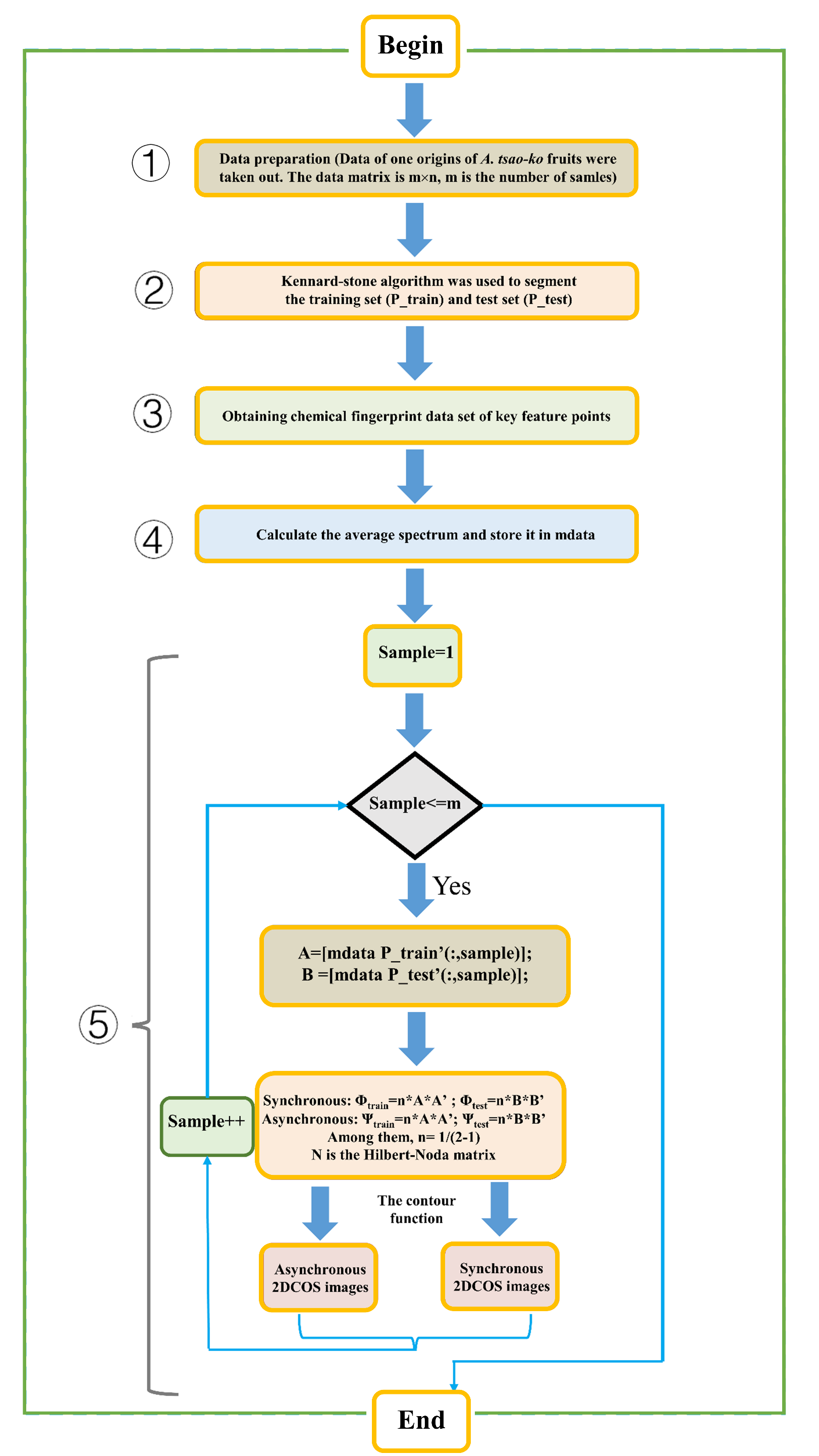


**Figure S2.** A flowchart of whole conversion processes of 2DOCS images through matlab2017b.


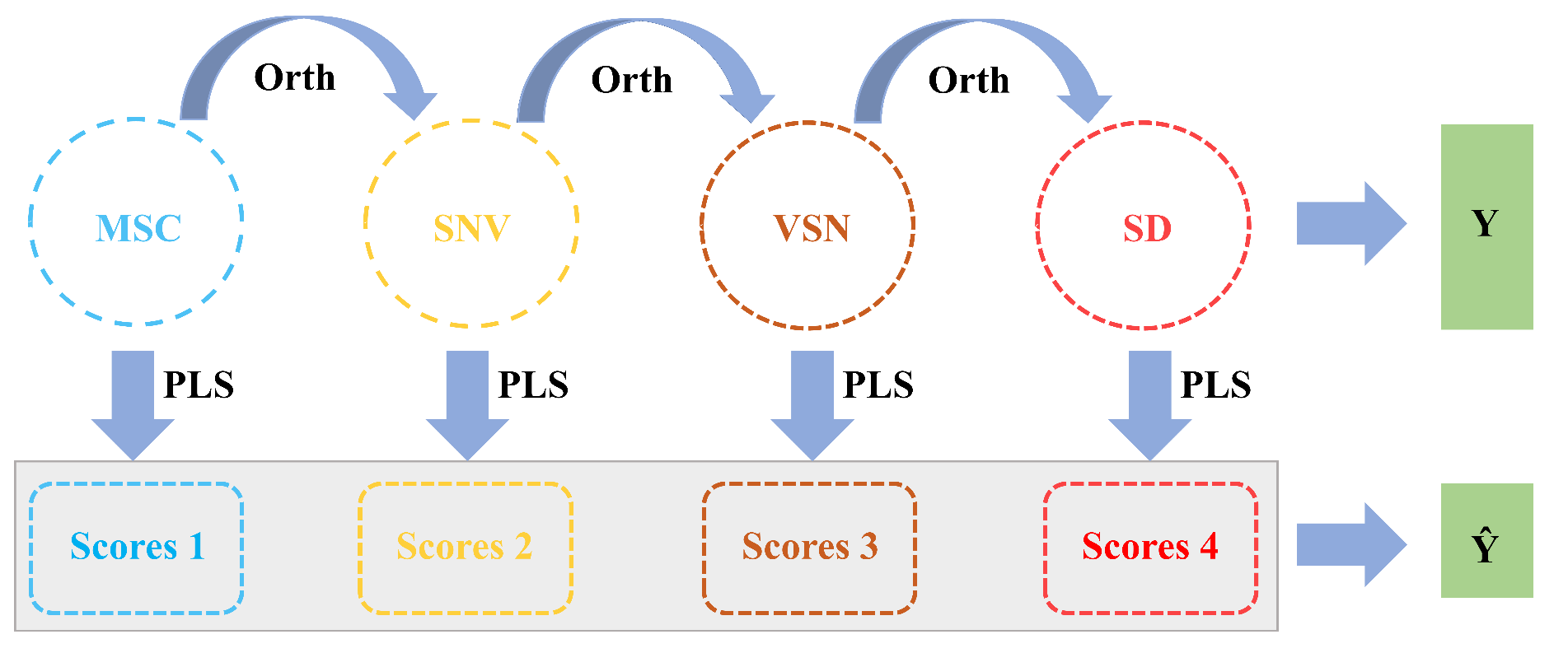


**Figure S3.** The schematic of SPORT for sequential fusion of pre-processing methods.


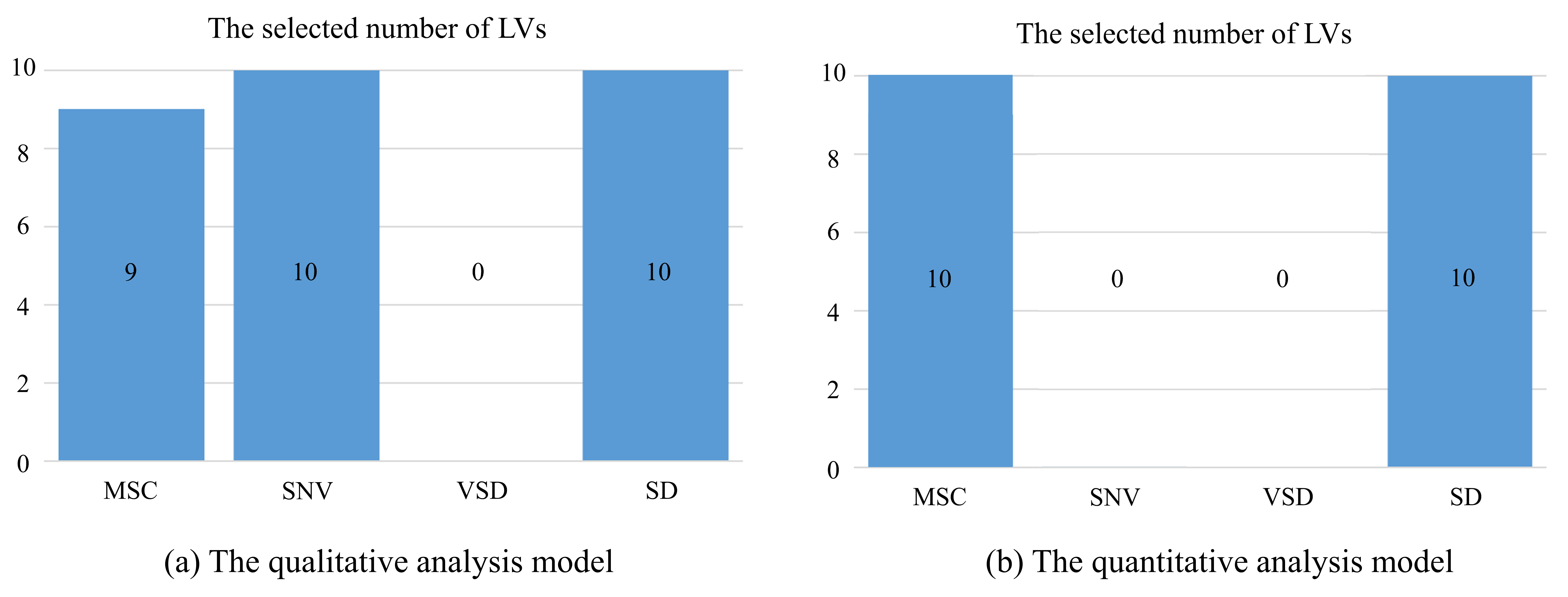


**Figure S4.** The number of selected LVs in SPORT model. (a) the qualitative analysis model; (b) the quantitative analysis model


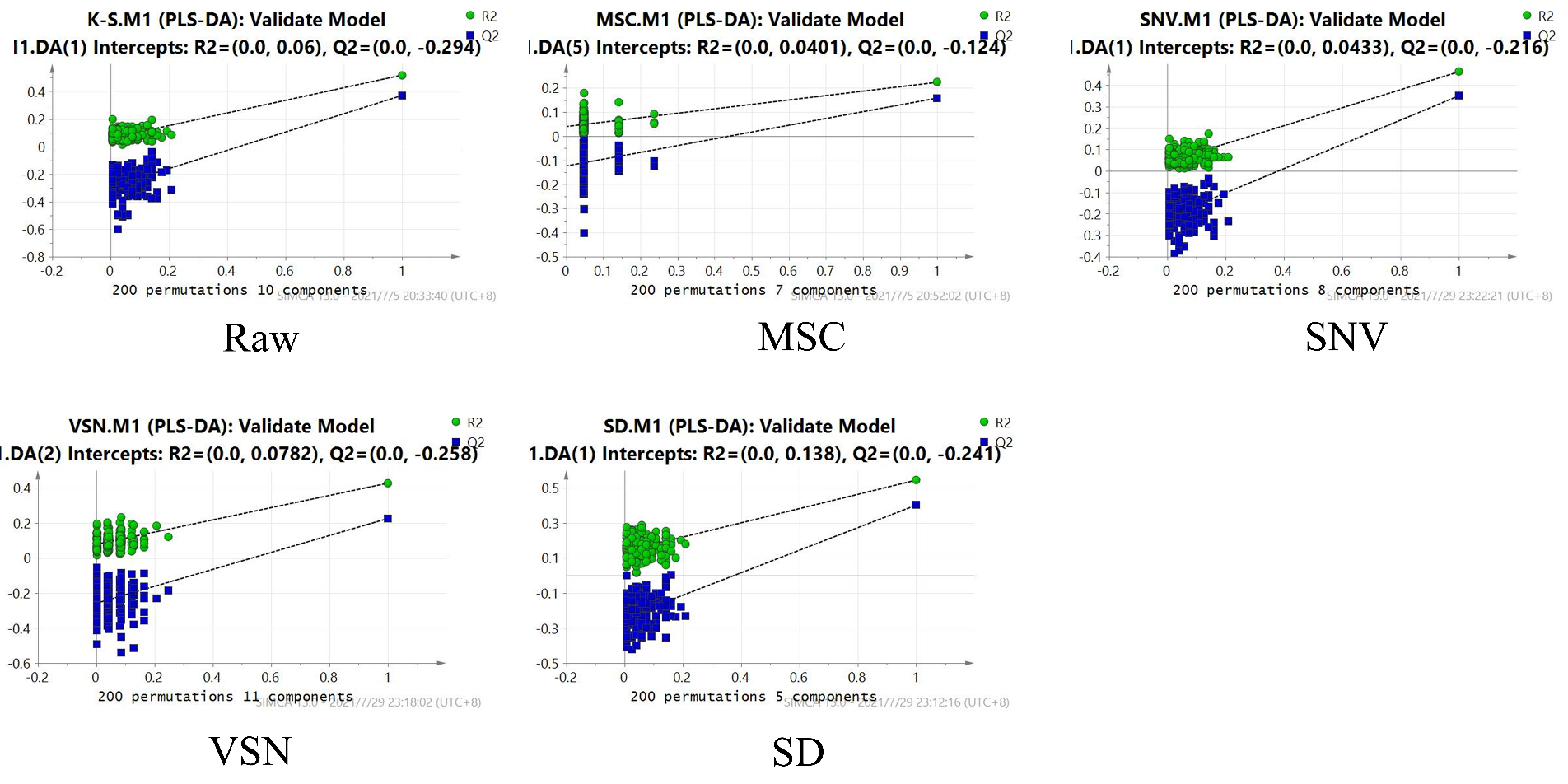


**Figure S5.** Permutation test results of five PLS-DA models.


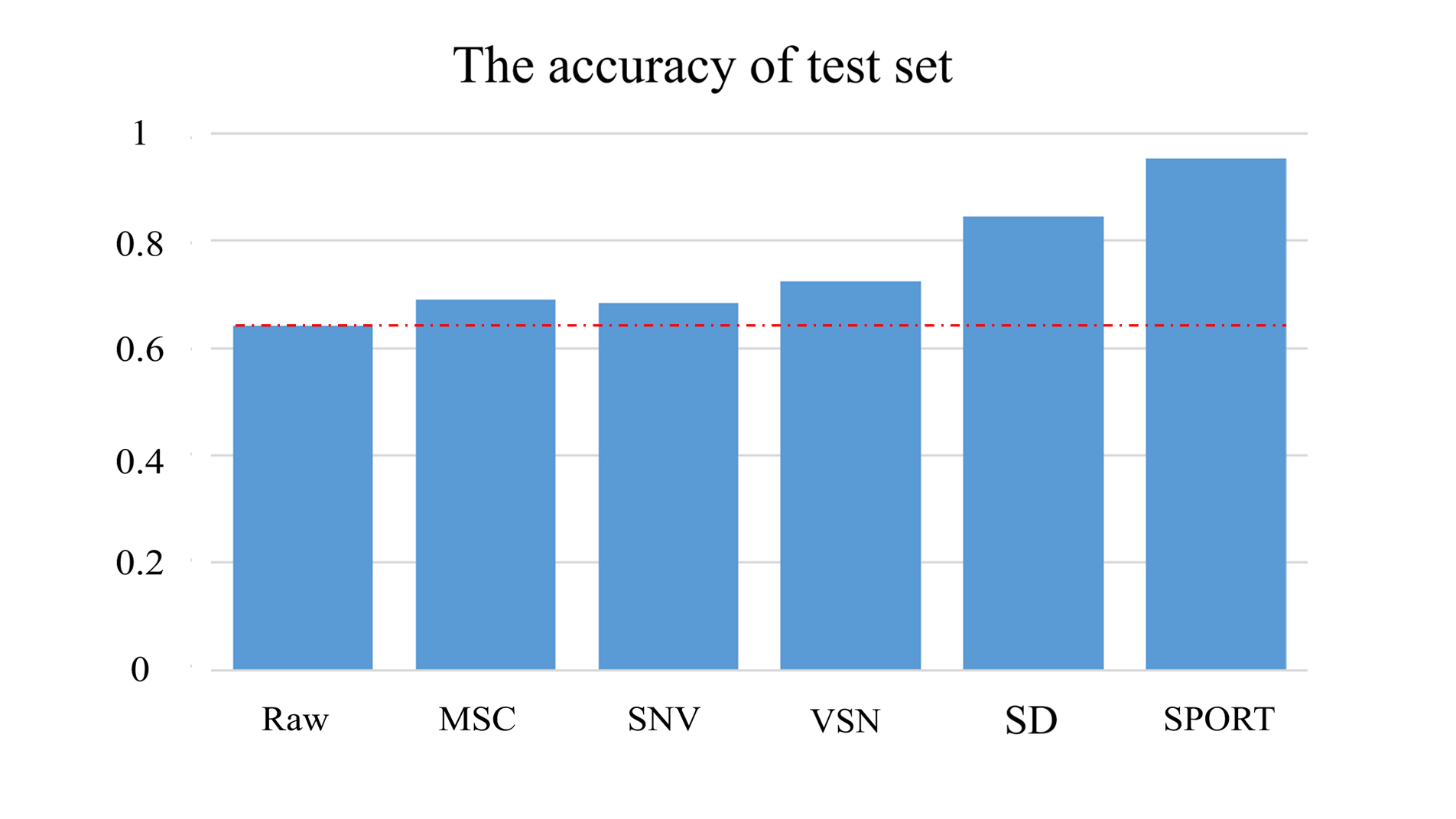


**Figure S6.** The accuracy of PLS-DA models.


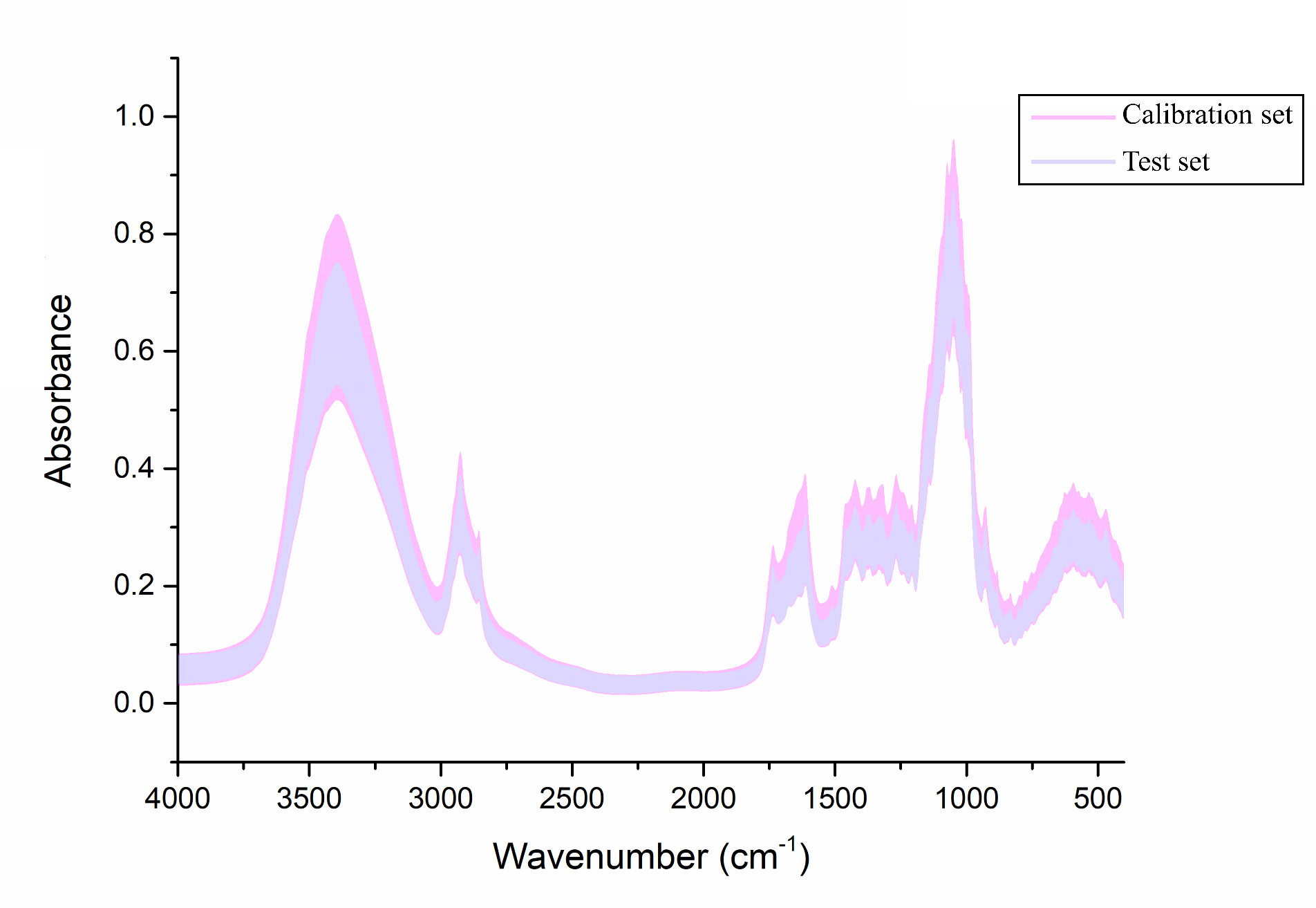


**Figure S7.** The distribution curve of the calibration set and test set.

**Table Captions:**

**Table S1.** The validation results of the method methodology.

**Table S2.** The RSD value for precision, stability, and repeatability in the methodology validation experiment.

**Table S3.** The content of total secoiridoids.

**Table S4.** The parameters of PLS-DA models.

**Table S5.** The confusion matrixes of PLS-DA models.

| Analyte | Regression equation | Linearity range (mg/g) | r^2^ | LOD (mg/g) | LOQ (mg/g) |
| --- | --- | --- | --- | --- | --- |
| Gentiopicroside | Y=5826.569X + 130.945 | 0.0040-0.7998 | 0.9991 | 0.0347 | 0.1158 |
| Swertiamarin | Y=7976.8679X+25.1673 | 0.0018-0.7683 | 0.9999 | 0.0014 | 0.0046 |
| Sweroside | Y=4250.2396X-2.0569 | 0.0018-0.7001 | 0.9999 | 0.0018 | 0.0060 |

**Table S1.** The validation results of the method methodology.

| Analyte | Precision RSD (%) | | | | Stability RSD (%) | Repeatability RSD (%) |
| --- | --- | --- | --- | --- | --- | --- |
|  | Inter-day | | | Intra-day |  |  |
| Gentiopicroside | 0.81 | 0.02 | 0.08 | 0.06 | 0.91 | 0.99 |
| Swertiamarin | 0.93 | 0.18 | 0.85 | 1.08 | 1.15 | 0.61 |
| Sweroside | 3.06 | 1.48 | 3.15 | 1.14 | 1.25 | 4.49 |

**Table S2.** The RSD value for precision, stability, and repeatability in the methodology validation experiment.

| No. | The content of total secoiridoids |
| --- | --- |
| 1 | 55.02 |
| 2 | 2.96 |
| 3 | 47.50 |
| 4 | 65.30 |
| 5 | 37.92 |
| 6 | 29.44 |
| 7 | 43.65 |
| 8 | 30.51 |
| 9 | 37.21 |
| 10 | 41.32 |
| 11 | 48.90 |
| 12 | 23.03 |
| 13 | 49.15 |
| 14 | 49.88 |
| 15 | 37.25 |
| 16 | 41.30 |
| 17 | 42.89 |
| 18 | 61.76 |
| 19 | 54.03 |
| 20 | 29.59 |
| 21 | 24.78 |
| 22 | 21.71 |
| 23 | 44.30 |
| 24 | 42.16 |
| 25 | 46.08 |
| 26 | 38.11 |
| 27 | 81.15 |
| 28 | 53.97 |
| 29 | 50.35 |
| 30 | 60.33 |
| 31 | 51.02 |
| 32 | 34.11 |
| 33 | 40.25 |
| 34 | 14.28 |
| 35 | 71.52 |
| 36 | 34.49 |
| 37 | 43.75 |
| 38 | 50.16 |
| 39 | 28.08 |
| 40 | 52.79 |
| 41 | 65.96 |
| 42 | 68.23 |
| 43 | 44.25 |
| 44 | 43.99 |
| 45 | 56.85 |
| 46 | 37.21 |
| 47 | 40.95 |
| 48 | 39.35 |
| 49 | 46.53 |
| 50 | 41.73 |
| 51 | 38.78 |
| 52 | 44.88 |
| 53 | 48.63 |
| 54 | 48.49 |
| 55 | 65.03 |
| 56 | 29.43 |
| 57 | 50.11 |
| 58 | 40.79 |
| 59 | 42.74 |
| 60 | 44.06 |
| 61 | 35.99 |
| 62 | 37.88 |
| 63 | 44.13 |
| 64 | 66.92 |
| 65 | 24.70 |
| 66 | 39.02 |
| 67 | 64.69 |
| 68 | 46.44 |
| 69 | 26.37 |
| 70 | 49.66 |
| 71 | 63.54 |
| 72 | 31.32 |
| 73 | 38.26 |
| 74 | 44.99 |
| 75 | 35.76 |
| 76 | 42.35 |
| 77 | 49.39 |
| 78 | 59.12 |
| 79 | 49.09 |
| 80 | 38.34 |
| 81 | 53.91 |
| 82 | 31.45 |
| 83 | 32.89 |
| 84 | 83.04 |
| 85 | 49.99 |
| 86 | 38.18 |
| 87 | 56.68 |
| 88 | 47.33 |
| 89 | 27.63 |
| 90 | 51.47 |
| 91 | 46.49 |
| 92 | 76.69 |
| 93 | 53.78 |
| 94 | 31.80 |
| 95 | 57.08 |
| 96 | 41.05 |
| 97 | 49.90 |
| 98 | 60.20 |
| 99 | 52.05 |
| 100 | 38.11 |
| 101 | 41.28 |
| 102 | 56.21 |
| 103 | 37.66 |
| 104 | 43.37 |
| 105 | 28.04 |
| 106 | 53.78 |
| 107 | 42.63 |
| 108 | 46.88 |
| 109 | 48.81 |
| 110 | 61.58 |
| 111 | 43.73 |
| 112 | 49.42 |
| 113 | 55.51 |
| 114 | 63.13 |
| 115 | 68.94 |
| 116 | 16.01 |
| 117 | 43.76 |
| 118 | 67.14 |
| 119 | 51.39 |
| 120 | 47.70 |
| 121 | 51.19 |
| 122 | 34.25 |
| 123 | 51.26 |
| 124 | 52.83 |
| 125 | 34.99 |
| 126 | 62.11 |
| 127 | 63.71 |
| 128 | 40.14 |
| 129 | 49.91 |
| 130 | 47.42 |
| 131 | 46.43 |
| 132 | 56.74 |
| 133 | 78.63 |
| 134 | 41.79 |
| 135 | 43.29 |
| 136 | 43.66 |
| 137 | 50.51 |
| 138 | 36.00 |
| 139 | 38.54 |
| 140 | 35.51 |
| 141 | 44.31 |
| 142 | 40.73 |
| 143 | 48.75 |
| 144 | 41.88 |
| 145 | 31.03 |
| 146 | 56.27 |
| 147 | 41.72 |
| 148 | 24.16 |
| 149 | 36.83 |
| 150 | 50.51 |
| 151 | 34.33 |
| 152 | 59.33 |
| 153 | 75.29 |
| 154 | 43.78 |
| 155 | 58.78 |
| 156 | 36.95 |
| 157 | 48.49 |
| 158 | 39.68 |
| 159 | 41.52 |
| 160 | 44.52 |
| 161 | 36.72 |
| 162 | 31.09 |
| 163 | 43.76 |
| 164 | 67.84 |
| 165 | 48.65 |
| 166 | 54.14 |
| 167 | 49.74 |
| 168 | 40.74 |
| 169 | 47.94 |
| 170 | 38.06 |
| 171 | 61.38 |
| 172 | 56.25 |
| 173 | 35.56 |
| 174 | 50.30 |
| 175 | 33.45 |
| 176 | 40.67 |
| 177 | 38.37 |
| 178 | 32.61 |
| 179 | 48.14 |
| 180 | 45.40 |
| 181 | 20.19 |
| 182 | 36.44 |
| 183 | 45.10 |
| 184 | 23.69 |
| 185 | 38.81 |
| 186 | 45.62 |
| 187 | 70.50 |
| 188 | 63.62 |
| 189 | 54.00 |
| 190 | 53.33 |
| 191 | 57.72 |
| 192 | 61.32 |
| 193 | 50.00 |
| 194 | 55.06 |
| 195 | 60.44 |
| 196 | 64.10 |
| 197 | 61.48 |
| 198 | 54.47 |
| 199 | 60.23 |
| 200 | 44.85 |
| 201 | 48.49 |
| 202 | 50.74 |
| 203 | 60.04 |
| 204 | 57.21 |
| 205 | 54.51 |
| 206 | 43.88 |
| 207 | 48.76 |
| 208 | 61.94 |
| 209 | 34.85 |
| 210 | 44.47 |
| 211 | 32.61 |
| 212 | 32.76 |
| 213 | 35.14 |
| 214 | 31.67 |
| 215 | 35.43 |
| 216 | 38.38 |
| 217 | 32.99 |
| 218 | 47.77 |
| 219 | 38.79 |
| 220 | 43.25 |
| 221 | 38.15 |
| 222 | 59.23 |
| 223 | 52.32 |
| 224 | 47.82 |
| 225 | 39.00 |
| 226 | 50.37 |
| 227 | 48.44 |
| 228 | 49.19 |
| 229 | 40.09 |
| 230 | 45.16 |
| 231 | 48.15 |
| 232 | 47.56 |
| 233 | 50.43 |
| 234 | 51.97 |
| 235 | 62.63 |
| 236 | 45.21 |
| 237 | 39.12 |
| 238 | 53.09 |
| 239 | 46.34 |
| 240 | 57.66 |
| 241 | 57.01 |
| 242 | 41.77 |
| 243 | 48.73 |
| 244 | 47.87 |
| 245 | 45.79 |
| 246 | 40.27 |
| 247 | 42.41 |
| 248 | 52.08 |
| 249 | 58.24 |
| 250 | 44.05 |
| 251 | 38.69 |
| 252 | 32.29 |
| 253 | 30.73 |
| 254 | 42.82 |
| 255 | 46.15 |
| 256 | 39.97 |
| 257 | 43.29 |
| 258 | 44.02 |
| 259 | 40.19 |
| 260 | 35.78 |
| 261 | 53.69 |
| 262 | 36.78 |
| 263 | 43.26 |
| 264 | 48.24 |
| 265 | 50.47 |
| 266 | 45.09 |
| 267 | 45.58 |
| 268 | 41.08 |
| 269 | 33.35 |
| 270 | 44.26 |
| 271 | 45.92 |
| 272 | 31.29 |
| 273 | 36.55 |

**Table S3.** The content of total secoiridoids.

| Methods | LVs | R^2^ | Q^2^ | RMSECV | RMSEE | RMSEP |
| --- | --- | --- | --- | --- | --- | --- |
| Raw | 10 | 0.366 | 0.221 | 0.308 | 0.282 | 0.230 |
| MSC | 7 | 0.294 | 0.211 | 0.310 | 0.296 | 0.291 |
| SNV | 8 | 0.317 | 0.216 | 0.309 | 0.292 | 0.291 |
| VSN | 11 | 0.426 | 0.244 | 0.304 | 0.268 | 0.272 |
| SD | 5 | 0.413 | 0.299 | 0.292 | 0.268 | 0.261 |
| SPORT | 4 | 0.751 | 0.641 | 0.221 | 0.168 | 0.207 |

**Table S4.** The parameters of PLS-DA models.

|  | Training set | | | | | |  | Test set | | | | |
| --- | --- | --- | --- | --- | --- | --- | --- | --- | --- | --- | --- | --- |
| Raw |  | O1 | O2 | O3 | O4 | O5 |  | O1 | O2 | O3 | O4 | O5 |
|  | O1 | 110 | 0 | 1 | 9 | 0 |  | 55 | 0 | 0 | 6 | 0 |
|  | O2 | 4 | 20 | 2 | 1 | 0 |  | 1 | 11 | 0 | 1 | 0 |
|  | O3 | 3 | 7 | 15 | 1 | 0 |  | 2 | 1 | 9 | 2 | 0 |
|  | O4 | 20 | 0 | 1 | 33 | 0 |  | 17 | 0 | 0 | 11 | 0 |
|  | O5 | 0 | 0 | 2 | 1 | 8 |  | 2 | 0 | 0 | 0 | 5 |
| MSC |  | O1 | O2 | O3 | O4 | O5 |  | O1 | O2 | O3 | O4 | O5 |
|  | O1 | 105 | 1 | 2 | 12 | 0 |  | 52 | 0 | 0 | 9 | 0 |
|  | O2 | 11 | 9 | 6 | 1 | 0 |  | 8 | 3 | 1 | 1 | 0 |
|  | O3 | 4 | 6 | 14 | 2 | 0 |  | 2 | 1 | 9 | 2 | 0 |
|  | O4 | 17 | 1 | 0 | 36 | 0 |  | 12 | 0 | 0 | 16 | 0 |
|  | O5 | 3 | 0 | 3 | 4 | 1 |  | 4 | 0 | 1 | 2 | 0 |
| SNV |  | O1 | O2 | O3 | O4 | O5 |  | O1 | O2 | O3 | O4 | O5 |
|  | O1 | 105 | 1 | 1 | 13 | 0 |  | 54 | 0 | 0 | 7 | 0 |
|  | O2 | 10 | 11 | 5 | 1 | 0 |  | 6 | 4 | 2 | 1 | 0 |
|  | O3 | 3 | 6 | 16 | 1 | 0 |  | 3 | 0 | 9 | 2 | 0 |
|  | O4 | 17 | 1 | 0 | 36 | 0 |  | 14 | 0 | 0 | 14 | 0 |
|  | O5 | 1 | 0 | 2 | 4 | 4 |  | 1 | 0 | 1 | 2 | 3 |
| VSN |  | O1 | O2 | O3 | O4 | O5 |  | O1 | O2 | O3 | O4 | O5 |
|  | O1 | 113 | 0 | 0 | 7 | 0 |  | 59 | 0 | 0 | 2 | 0 |
|  | O2 | 3 | 22 | 2 | 0 | 0 |  | 1 | 11 | 0 | 1 | 0 |
|  | O3 | 4 | 8 | 12 | 2 | 0 |  | 2 | 5 | 5 | 2 | 0 |
|  | O4 | 15 | 0 | 0 | 39 | 0 |  | 15 | 0 | 0 | 13 | 0 |
|  | O5 | 1 | 0 | 1 | 4 | 5 |  | 4 | 0 | 0 | 2 | 1 |
| SD |  | O1 | O2 | O3 | O4 | O5 |  | O1 | O2 | O3 | O4 | O5 |
|  | O1 | 107 | 0 | 0 | 12 | 1 |  | 57 | 0 | 0 | 4 | 0 |
|  | O2 | 1 | 24 | 1 | 1 | 0 |  | 0 | 11 | 1 | 1 | 0 |
|  | O3 | 6 | 5 | 14 | 1 | 0 |  | 1 | 0 | 11 | 1 | 1 |
|  | O4 | 9 | 0 | 0 | 45 | 0 |  | 7 | 0 | 0 | 21 | 0 |
|  | O5 | 1 | 0 | 0 | 5 | 5 |  | 1 | 0 | 0 | 2 | 4 |
| SPORT |  | O1 | O2 | O3 | O4 | O5 |  | O1 | O2 | O3 | O4 | O5 |
|  | O1 | 119 | 0 | 0 | 1 | 0 |  | 60 | 0 | 0 | 1 | 0 |
|  | O2 | 0 | 27 | 0 | 0 | 0 |  | 0 | 13 | 0 | 0 | 0 |
|  | O3 | 0 | 0 | 26 | 0 | 0 |  | 0 | 2 | 12 | 0 | 0 |
|  | O4 | 0 | 0 | 0 | 53 | 1 |  | 1 | 0 | 0 | 27 | 0 |
|  | O5 | 0 | 0 | 0 | 0 | 11 |  | 1 | 0 | 0 | 0 | 6 |

**Table S5.** The confusion matrixes of PLS-DA models.
